# Cancer Risk in Hashimoto’s Thyroiditis: a Systematic Review and Meta-Analysis

**DOI:** 10.3389/fendo.2022.937871

**Published:** 2022-07-12

**Authors:** Xiaojie Hu, Xuanyu Wang, Yue Liang, Xin Chen, Siyuan Zhou, Wenting Fei, Yuxin Yang, Huafa Que

**Affiliations:** ^1^ Department of Traditional Chinese Surgery, Longhua Hospital Shanghai University of Traditional Chinese Medicine, Shanghai, China; ^2^ Shanghai University of Traditional Chinese Medicine, Shanghai, China

**Keywords:** Hashimoto’s thyroiditis, cancer risk, observational study, systematic review, meta-analysis

## Abstract

**Objective:**

Research data suggest that patients with Hashimoto’s thyroiditis may increase the risk of cancer. However, existing research is inconsistent with this view. Therefore, to investigate the effect of Hashimoto’s thyroiditis on the risk of developing cancer, we conducted this study.

**Methods:**

We searched the PubMed and Embase databases from database establishment until March 2022. After rigorous literature screening by two authors, 23 studies that met the inclusion criteria were identified, and the required data were independently extracted.

**Results:**

We retrieved 3591 records, and after the screening, 11 case-control studies and 12 cohort studies were included in the analysis. Data analysis suggested that patients with Hashimoto’s thyroiditis had an increased risk of developing breast cancer, urogenital cancer, digestive organs cancer, hematologic cancer, and a low risk of respiratory cancers.

**Conclusions:**

This systematic review and meta-analysis showed that patients with HT may have a significantly increased risk of thyroid cancer, breast cancers, lung cancer, digestive system cancer, urogenital cancers, blood cancers, and prolactinoma people without HT.

**Systematic Review Registration:**

https://www.crd.york.ac.uk/prospero/, identifier CRD 42022320741.

## 1 Introduction

Hashimoto’s thyroiditis (HT) is the most frequent autoimmune disease, also known as chronic lymphocytic or autoimmune thyroiditis, which often manifests clinically as enlarged thyroid, lymphocytic infiltration, and increased autoimmune antibodies (1, 2). It is also a disease of autoimmune aseptic inflammation. Research shows that chronic inflammation is an indispensable participant in cancer development (3–7). Therefore, more and more research institutes begin to study the relationship between HT and cancer. However, controversy over whether HT and cancer development are related as research increases. Recently, a case-control study showed that abnormal thyroid function was associated with the development of rectal cancer (8). A 22-year study demonstrates that patients with HT developing papillary thyroid carcinoma are more likely to develop multifocal tumors (9). Consistent with this, some studies show that HT and the occurrence of thyroid cancer have a strong correlation (10, 11). Unlike this, several studies indicate that HT is not associated with the development of thyroid cancer and breast cancer (12, 13). Given conflicting evidence and newly added epidemiological studies, we conducted a meta-study to examine and assess the association between HT and cancer.

## 2 Methods

### 2.1 Registration

This review was carried out following PRISMA and registered with PROSPERO (CRD 42022320741).

### 2.2 Search Strategy

The literature search was conducted according to the principles recommended in the Preferred Reporting Project for Systematic Reviews and Meta-Analysis (PRISMA). Two authors (Wenting Fei and Yuxin Yang) independently searched PubMed and Embase databases by combining search terms with free words: “Hashimoto Disease”, OR “Disease, Hashimoto”, OR “Autoimmune thyroiditis”, OR “Hashimoto Struma”, OR “Hashimoto Thyroiditis”, OR “Hashimoto Thyroiditides”, OR “Thyroiditides, Hashimoto”, OR “Thyroiditis, Hashimoto”, OR “Hashimoto’s Syndrome”, OR “Hashimoto Syndrome”, OR “Hashimoto’s Syndromes”, OR “Hashimotos Syndrome”, OR “Syndrome, Hashimoto’s”, OR “Syndromes, Hashimoto’s”, OR “Hashimoto’s Struma”, OR “Chronic Lymphocytic Thyroiditis”, OR “Chronic Lymphocytic Thyroiditides”, OR “Lymphocytic Thyroiditides, Chronic”, OR”Lymphocytic Thyroiditis, Chronic”, OR “Thyroiditides, Chronic Lymphocytic”, OR “Thyroiditis, Chronic Lymphocytic”, OR “Hashimoto’s Disease”, OR “Disease, Hashimoto’s”, OR “Hashimotos Disease”; “Neoplasms”, OR “Tumor”, OR “Neoplasm”, OR “Tumors”, OR “Neoplasia”, “Neoplasias”, OR “Cancer”, OR “Cancers”, OR “Malignant Neoplasm”, OR “Malignancy”, “Malignancies”, OR “Malignant Neoplasms”, OR “Neoplasm, Malignant”, OR “Neoplasms, Malignant”, OR “Benign Neoplasms”, OR “Benign Neoplasm”, OR “Neoplasms, Benign”, OR “Neoplasm, Benign”. All articles were published in English from the establishment of the museum to March 2022.

### 2.3 Inclusion and Exclusion Criteria

The inclusion criteria for this study are as follows: (i) the study type must be observational; (ii) the subject of the study is Hashimoto’s thyroiditis and cancer; (iii) the study participants must be adults (≥18 years old) regardless of gender or race. The exclusion criteria were as follows: (i) the type of study design was not observational; (ii) reviews, case reports, and animal studies; (iii) the study information was incomplete and the authors could not be contacted to extract the information needed for this study data information. According to these criteria, two authors (Xin Chen and Siyuan Zhou) provided the titles and abstracts of the readings for screening, followed by full-text reading, excluded studies that did not meet the inclusion requirements, and finally screened out eligible articles. Inconsistencies arising from the review process were resolved with the help of a third author (Huafa Que).

### 2.4 Study Selection

**Figure 1:**
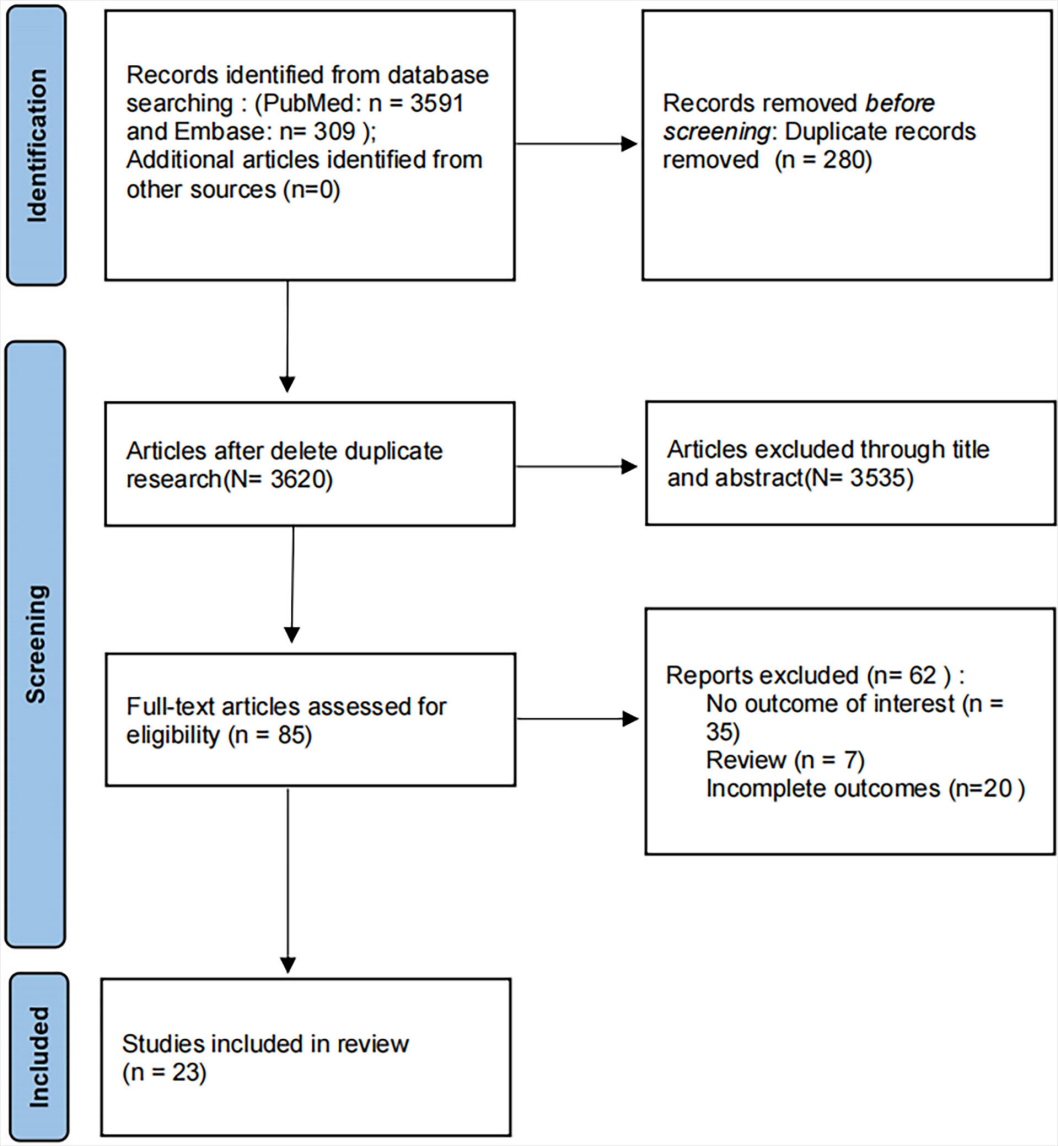
Flow diagram of systemic review procedure.

A total of 3591 documents were retrieved, 280 duplicate documents were excluded, and 3620 documents were carried out for further research. 3535 articles were excluded through title and abstract due they did not meet the inclusion criteria. The full-text articles 85 were evaluated for eligibility. Finally, 23 papers were included (**Figure 1**).

### 2.5 Assessment of Bias Risk

The methodological quality of all included articles was assessed by two authors (Xiaojie Hu and Xuanyu Wang) independently using the Newcastle-Ottawa Scale (NOS) (14). The NOS scale evaluation items include 8 items, and all literature quality evaluation scores are 7 points or above. All disagreements encountered were discussed and adjudicated by a third senior author (Huafa Que).

### 2.6 Data Extraction

For the included literature, we extracted the following information: first author, year of publication, country, study design, the sample of the case, the sample of controls, duration of the study, odds ratios (OR) or relative risks (RR) with 95% confidence intervals (Cl), and outcomes. We categorized cancer types in literature studies by the site of disease, including thyroid cancer, digestive organs cancer(colorectal cancer, stomach, hepatoma), genitourinary cancer (uterus, cervical, ovary, prostate, bladder, kidney), breast cancer, respiratory cancer (lung cancer), prolactinoma, and leukemia. Data were extracted independently by three authors (Xiaojie Hu, Xuanyu Wang, and Yue Liang) and reviewed by one of them (Xiaojie Hu) to ensure the accuracy of the data extraction.

### 2.7 Data Analyses

For dichotomous data, we used OR or RR with 95% confidence intervals (Cl). For continuous data, we use the weighted mean difference with 95% CI. ORs are used to describe case-control studies, while RRs are used to describe cohort studies. Heterogeneity between included studies was assessed using the Q-test and the squared value of I (15, 16). In the Q-test, a P-value < 0.10 or I squared > 50% indicated a specific statistical significance of heterogeneity between studies, and a random-effects model was used. Conversely, the square of I was ≤50%, suggesting that the heterogeneity among the included studies was small, and a fixed-effects model could be used. Subgroup analyses and sensitivity analyses were used to explore the reasons for heterogeneity. Egger’s test and funnel plot were used to analyze the possibility of publication bias. Therefore, STATA 16.0 software was used for statistical analysis.

### 2.8 Ethical Approval

This study does not involve the examination of the participants and therefore does not require ethical approval.

## 3 Result

### 3.1 Study Characteristics

The 3591 articles on the association between Hashimoto’s thyroiditis and thyroid cancer were screened, and 3620 were retrieved after the removal of repeated documents. Browsing full-text articles assessed for initial screening literature, 3535 literature were excluded due to they failed to meet inclusion criteria. Therefore, 23 articles (17–39) were included involving 11 case-controls and 12 cohorts. This study was incorporated The United States (7 studies), China (5 studies), Turkey (4 studies), Japan (1 study), Poland (1 study), Greece (1 study), Sri Lanka (1 study), Italy (1 study), Croatia (1 study), Bulgaria (1 study). The studies were performed in 10 regions and included 12917 cases and 60509 control subjects. Eleven of the 22 articles recorded the duration of the study, with the longest being 22 years and the shortest being 1 year (**Table 1**).

**Table 1:** Characteristics of included studies.

Study	Year of publication	Country	Study design	Sample of Cases	Sample of Controls	Duration of study, years	OR or RR (95% CI)	Outcomes
Siriweera (17)	2010	Sri Lanka	Case-control	349	2336	–	–	Thyroid cancer
Paparodis (18)	2014	USA	Case-control	567	2151	–	1.93 (1.60,2.34)	Thyroid cancer
Buyukasik (19)	2011	Turkey	case-control	77	840	–	2.24 (1.22,4.11)	Thyroid cancer
Zhang (20)	2014	China	Case-control	108	539	–	3.02 (1.94,4.69)	Thyroid cancer
Paparodis (21)	2019	USA	Cohort	617	3292	18	1.64 (1.38,1.95)	Thyroid cancer
Repplinger (22)	2017	USA	Case-control	217	981	–	1.34 (0.96,1.85)	Thyroid cancer
Mazokopakis (23)	2010	Greece	Case-control	42	98	–	1.56 (0.68,3.58)	Thyroid cancer
Zhang Y (24)	2014	China	Case-control	835	7685	–	1.80 (1.53,2.11)	Thyroid cancer
Dubrarka (25)	2009	Croatia	Cohort	2156	8352	12	–	Thyroid cancer
Jackson (26)	2020	USA	Cohort	52	307	8.25	0.99 (0.65,1.50)	Thyroid cancer
Zhang (27)	2012	China	Case-control	653	5456	–	–	Thyroid cancer
Mukasa (28)	2010	Japan	Cohort	2036	1652	1	1.57 (1.38,1.78)	Thyroid cancer
Chen (30)	2013	China	Cohort	1521	6084	12	2.33 (0.92,5.92) 0.19 (0.01,3.25) 1.76 (0.96,3.23) 1.14 (0.38,3.47) 8.56 (0.78,94.37)	Thyroid cancer, Digestive organs cancer, Respiratory organs cancer, Breast cancer, Genitourinary cancer Hematologic cancer
Morais (31)	2019	USA	Cohort	2651	7200	22	1.39 (1.26,1.52)	Thyroid cancer
Holm (32)	1985	USA	Cohort	329	829	22	2.52 (0.36, 17.81) 17.64 (2.18,142.80) 1.51 (0.55,4.13) 1.96 (0.74,5.22) 2.60 (0.37,18.37)	Thyroid cancer, Digestive organs cancer, Respiratory organs cancer, Breast cancer, Female genitul organs, Hematologic cancer
Liu (33)	2014	China	Cohort	1328	5104	6	1.58 (1.45,1.71)	Thyroid cancer
Anil (34)	2010	Turkey	Cohort	191	713	3.25	0.40 (0.09,1.70)	Thyroid cancer
Gul (35)	2010	Turkey	Case-control	92	521	–	–	Thyroid cancer
Consorti (36)	2010	Italy	Cohort	69	335	13.17	1.44 (0.97,2.13)	Thyroid cancer
Konturek (37)	2013	Poland	Cohort	452	7093	8	–	Thyroid cancer
Dailey (38)	1954	USA	Cohort	205	208	10	1.16 (0.74,1.82)	Thyroid cancer
Dogansen (29)	2016	Turkey	Case-control	83	78	–	2.41 (1.14,5.11)	Prolactinoma
Elenkova (39)	2017	Bulgaria	Case-control	154	106	–	2.84 (1.41,5.72)	Prolactinoma

### 3.2 Study Quality

The risk of bias assessments was assessed through the Newcastle-Ottawa-Scale tool. The average NOS score is 7.66. All incorporated literature was included in the quality assessment, and all articles received a score of 5 or more, of which 1 received 9 points, 13 received 8 points (**Table 2**) and 9 received 7 points (**Table 3**). No significant publication bias was detected for all cancer risks.

**Table 2:** The quality assessment of 12 included studies based on the Newcastle–Ottawa Scale (range 0–9).

Study	Representativenessof the exposedcohort	Selection of thenon-exposedcohort	Ascertainment ofexposure	Demonstration thatoutcome of interestwas not present atstart of study	Comparability ofcohorts on the basisof the design oranalysis	Assessment of outcome	Was follow-uplong enough foroutcomes tooccur	Adequacy offollow up ofcohorts	Quality score
Paparodis (21)	*	*	*	*	**	*	*	*	9
Dubrarka (25)	*	*	*	*	*	*	*	*	8
Jackson (26)	*	*	*	*	*	*	*	*	8
Mukasa	*	*	*	*	*	*	*	*	8
Chen (30)	*	*	*	*	*	*	*	*	8
Morais (31)	*	*	*	*	*	*	*	*	8
Holm (32)	*	*	*	*	*	*	*	*	8
Liu (33)	*	*	*	*	*	*	*	*	8
Anil (34)	*	*	*	*	*	*	*	*	8
Consorti (36)	*	*	*	*	*	*	*	*	8
Konturek (37)	*	*	*	*	*	*	*	*	8
Dailey (38)	*	*	*	*	*	*	*	*	8

**Table 3:** The quality assessment of 12 included studies based on the Newcastle–Ottawa Scale (range 0–9).

Study	Adequacy of the case definitionrt	Representativeness of cases	Choice of controls	Definitionof Control	Comparability of case-control on the basis of the designor analysis	Exposure Investigationand Assessment Methods	Whether exposure of cases and controls was determined using the same method	non-response rate	Quality score
Siriweera (17)	*	*	/	*	*	*	*	*	7
Paparodis (18)	*	*	/	*	*	*	*	*	7
Buyukasik (19)	*	*	/	*	*	*	*	*	7
Zhang (20)	*	*	/	*	*	*	*	*	7
Repplinger (22)	*	*	/	*	*	*	*	*	7
Mazokopakis (23)	*	*	/	*	*	*	*	*	7
Zhang Y (24)	*	*	/	*	*	*	*	*	7
Zhang (27)	*	*	/	*	*	*	*	*	7
Gul (35)	*	*	/	*	*	*	*	*	7
Elenkova (39)	*	*	*	*	*	*	*	*	8
Dogansen (29)	*	*	/	*	*	*	*	*	7

### 3.3 Rate of Cancers in HT Patients

This study incorporates 11 case-controls and 12 cohorts, so we evaluate all included studies according to different experimental design types. For instance, we reported case-control results as OR and cohorts as RR. The research on HT patients referred to 13 human cancer types: thyroid cancer, breast cancer, lung cancer, stomach cancer, hepatoma cancer, colorectal cancer, uterus cancer, cervical cancer, ovary cancer, prostate cancer, bladder cancer, kidney cancer, and hematologic cancer. The relative risks/odds ratio of types of cancer among HT patients are listed in **Table 1**.

HT patients were reported to have a high cancer risk in referred cancers. The result of our meta-analysis displayed that the thyroid cancer rate of cancers in HT patients was the highest although some reported studies have shown that the association between thyroid cancer and HT is controversial (40–42). The rate of thyroid cancers in patients with HT from the 21 studies ranged from 0.61% to 58.43%, with a mean rate of 25.01%. The mean rate of breast cancer 1.40% (0.99%, 1.82%), respiratory organs cancer 1.06% (0, 2.15%), genitourinary cancer was 1.2% (0.3, 2.1), digestive organs cancer 2.21% (0.46%, 3.95%), and leukemia 0.37% (0.13%, 0.61%). Only one document mentioned malignant lymphoma, and 2 patients were found among 2036 HT patients. Among 329 HT patients, 3 patients of myeloma were found and no case was found in the control group.

### 3.4 Overall Cancer Risk in HT Patients

The literature describing thyroid cancer includes both case-control studies and cohort studies. To better analyze these data, therefore, we divided the literature into a more detailed division: OR values were used to describe case-control studies, while RR values were used to describe cohort studies.

**Figure 2:**
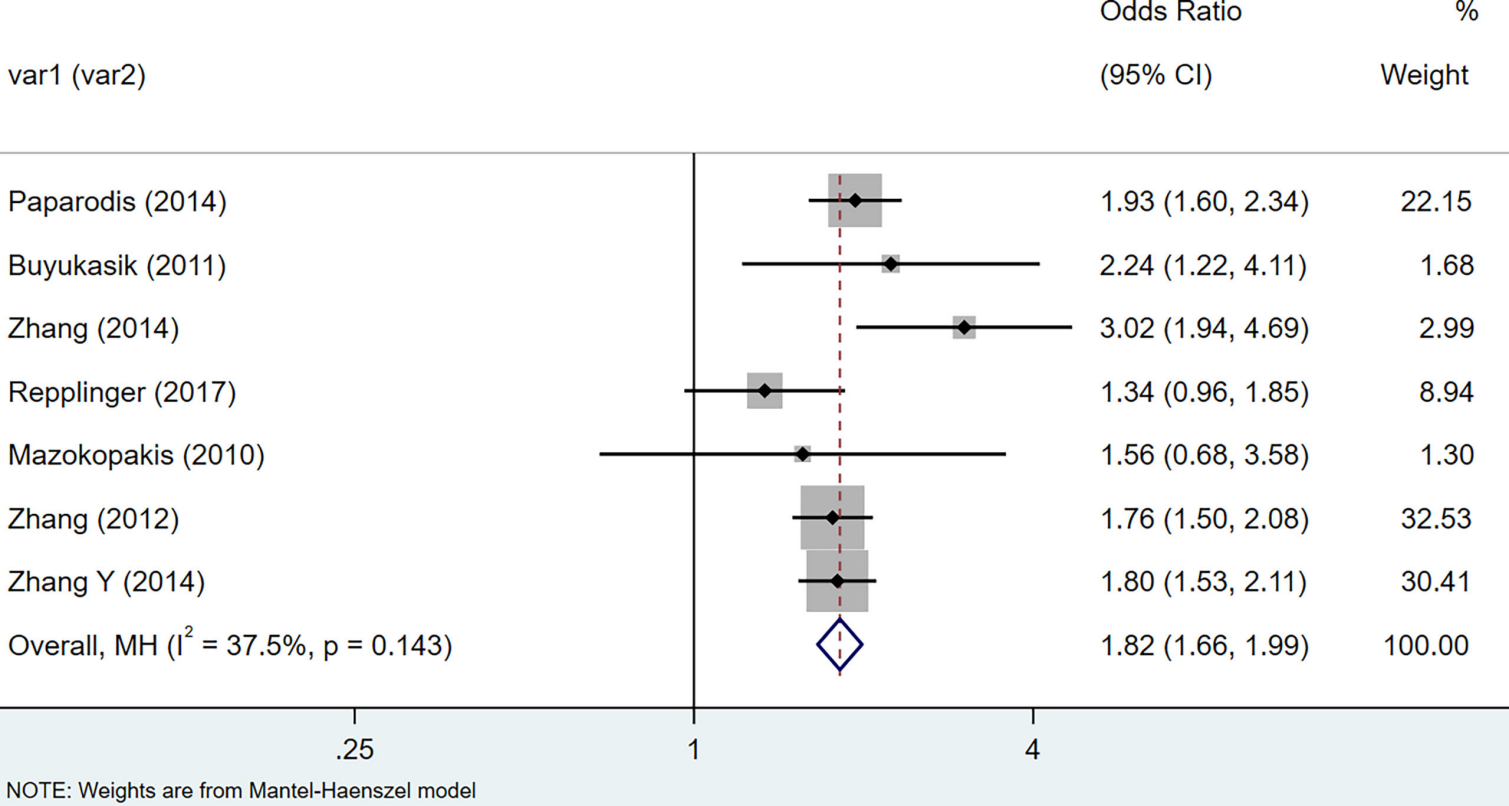
Forest plot of risk of thyroid cancer in patients with Hashimoto’s thyroiditis and those without Hashimoto’s thyroiditis in case-control studies.

In case-controls: Under the random-effects model, HT patients were reported to have a higher risk of the thyroid cancer (OR = 2.41, 95% CI = 1.81-3.20, I^2^ = 88.8%%, p < 0.0001). The 9 literature in this study were tested for heterogeneity, I squared >50%, p<0.1, suggesting that the heterogeneity among the literature selected in this study was statistically significant, consequently, sensitivity analysis was performed to find the reason. The results indicated that after removing studies (17) and (35), the combined effect size of the meta-analysis was large, so the two studies were removed and the study was conducted again. The test results indicated that there was no heterogeneity in the remaining 7 literature. After exclusion, a meta-analysis was performed using random-effects model (OR = 1.82, 95% CI = 1.66–1.99, I^2^ = 37.5%, p = 0.143), indicating that there was positive correlation between HT and thyroid cancer (**Figure 2**).

**Figure 3:**
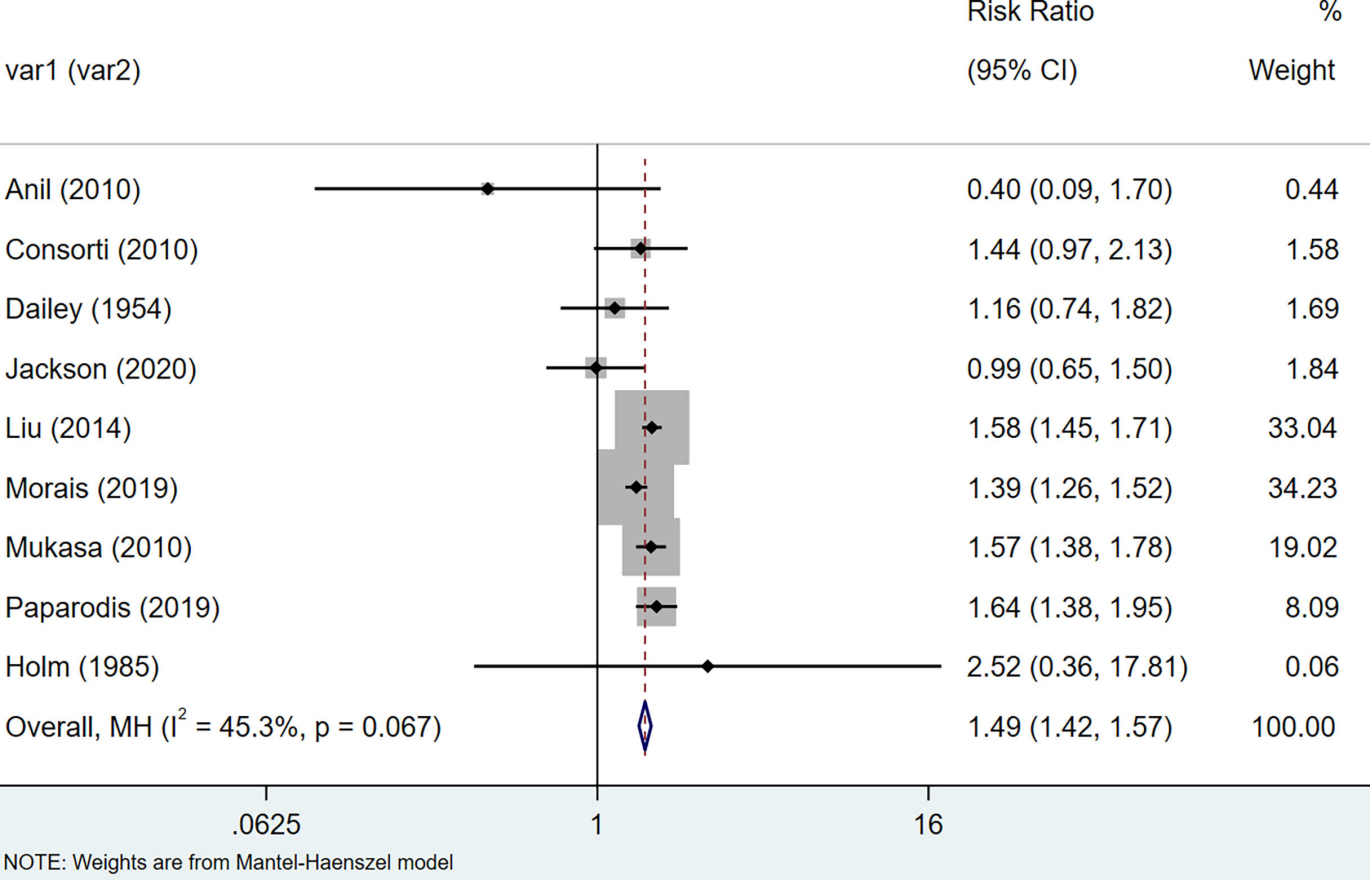
Forest plot of risk of thyroid cancer in patients with Hashimoto’s thyroiditis and those without Hashimoto’s thyroiditis in cohort studies.

In cohort: The results of the meta-analysis of thyroid cancer data extracted from 12 cohort studies suggest that the relative risks of thyroid cancer among HT patients are 1.62 (95% CI = 1.34–21.96, I^2^ = 90.0%, p < 0.001), showing that there was large heterogeneity among these studies, so we performed a sensitivity analysis. After removing the three articles (25, 30, 37) that had a greater impact on the study results, we re-used the fixed-effects model to conduct a meta-analysis, and the results suggested that compared with non-HT patients, the risk of HT patients with thyroid cancer increased by 0.49 times (RR = 1.49, 95% CI = 1.42–1.57, I^2^ = 45.3%, p = 0.067) (**Figure 3**). The results of the analysis were statistically significant.

**Figure 4:**
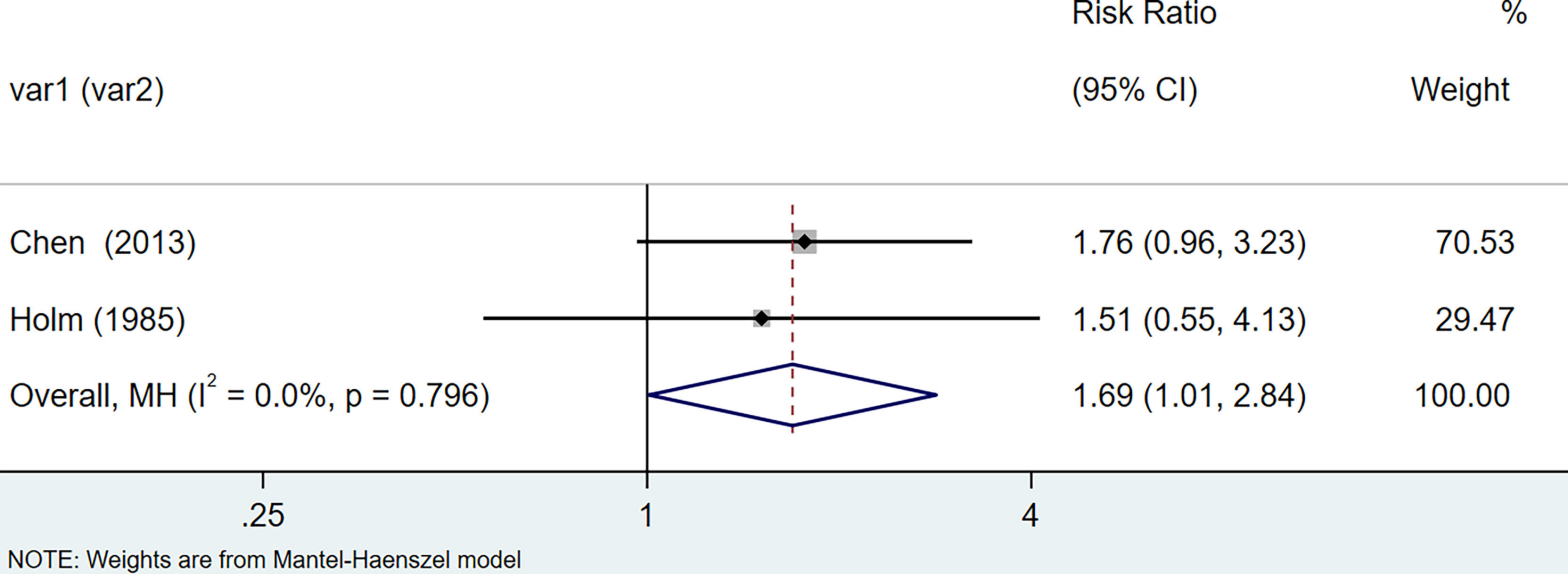
Forest plot of breast cancer risk in patients with Hashimoto’s thyroiditis and those without Hashimoto’s thyroiditis.

**Figure 5:**
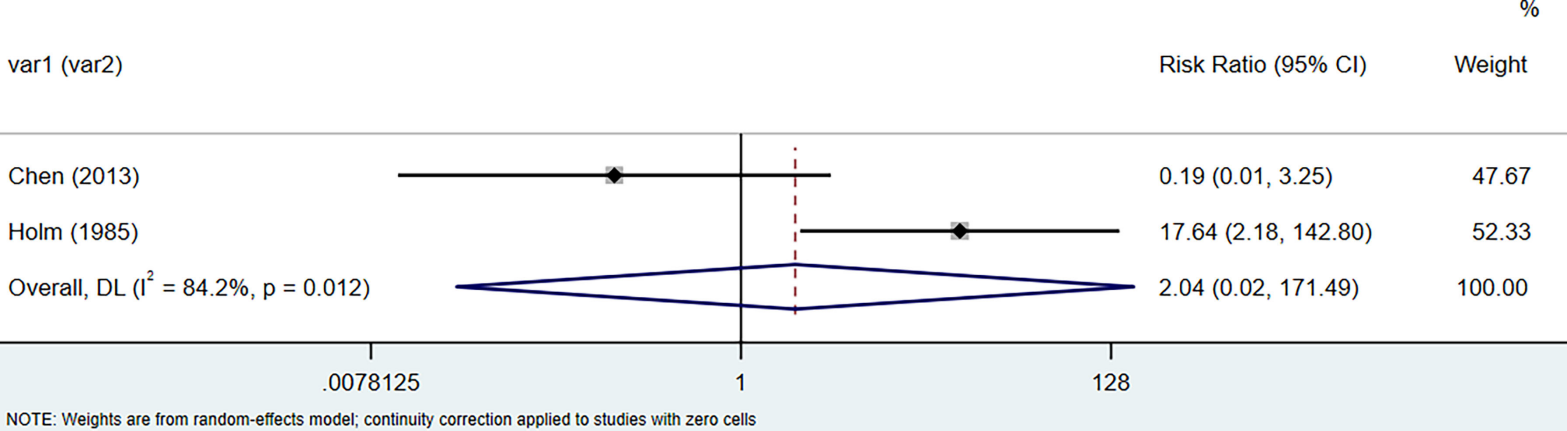
Forest plot of lung cancer risk in patients with Hashimoto’s thyroiditis and those without Hashimoto’s thyroiditis.

**Figure 6:**
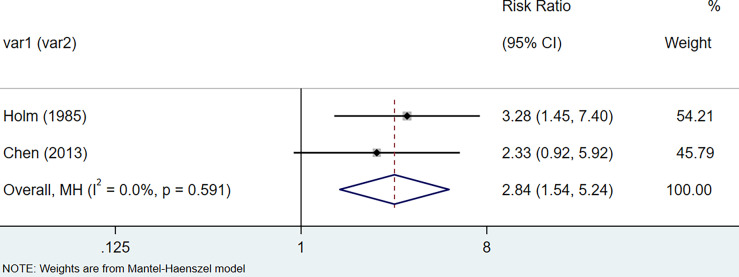
Forest plot of digestive organs cancer risk in patients with Hashimoto’s thyroiditis and those without Hashimoto’s thyroiditis.

**Figure 7:**
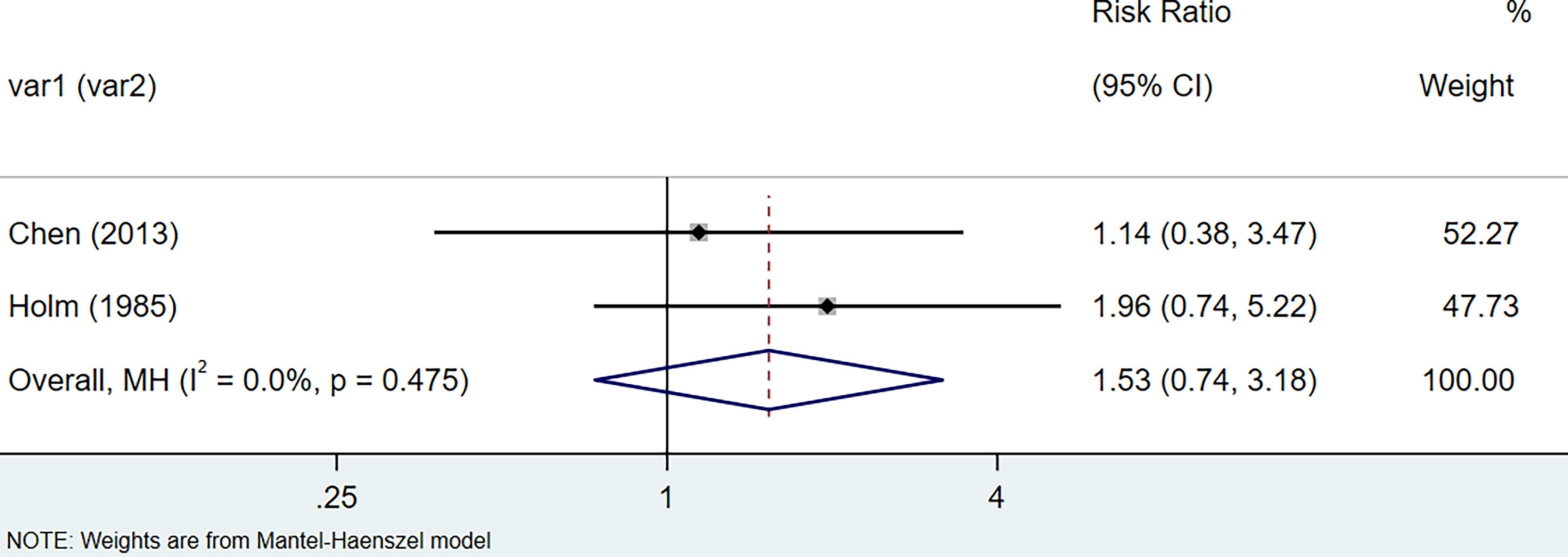
Forest plot of genitourinary cancer risk in patients with Hashimoto’s thyroiditis and those without Hashimoto’s thyroiditis.

**Figure 8:**
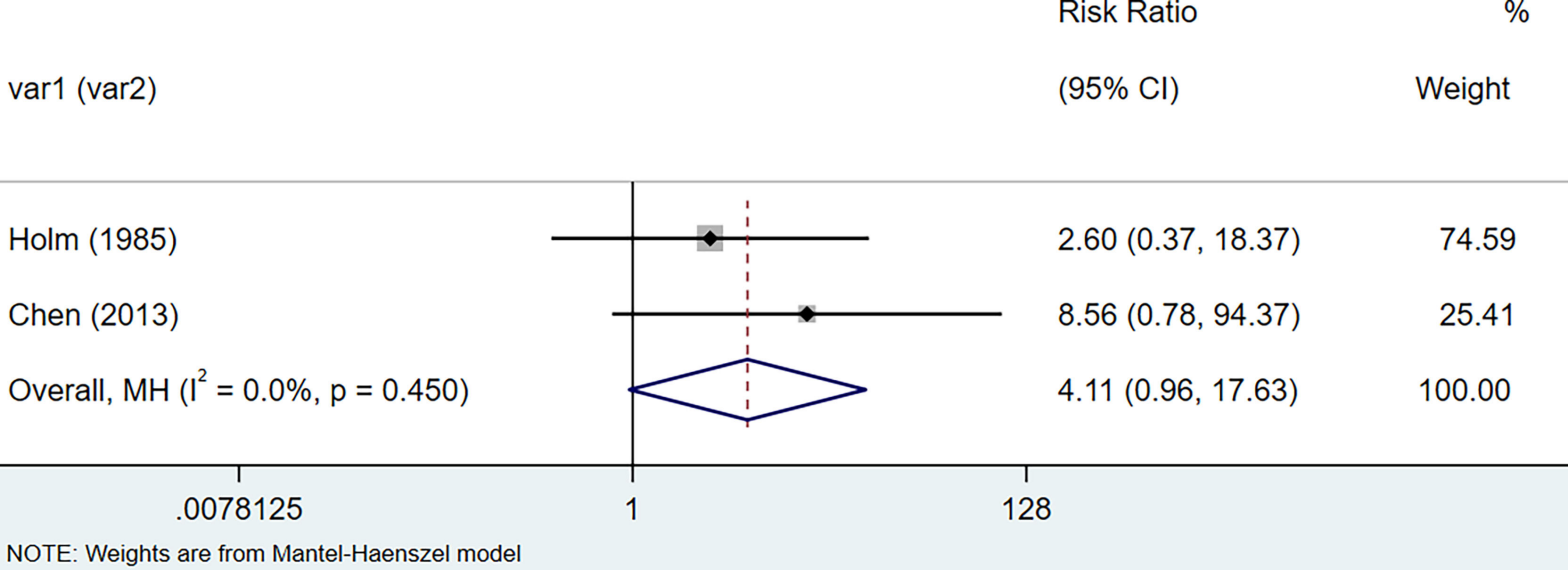
Forest plot of blood cancer risk in patients with Hashimoto’s thyroiditis and those without Hashimoto’s thyroiditis.

**Figure 9:**
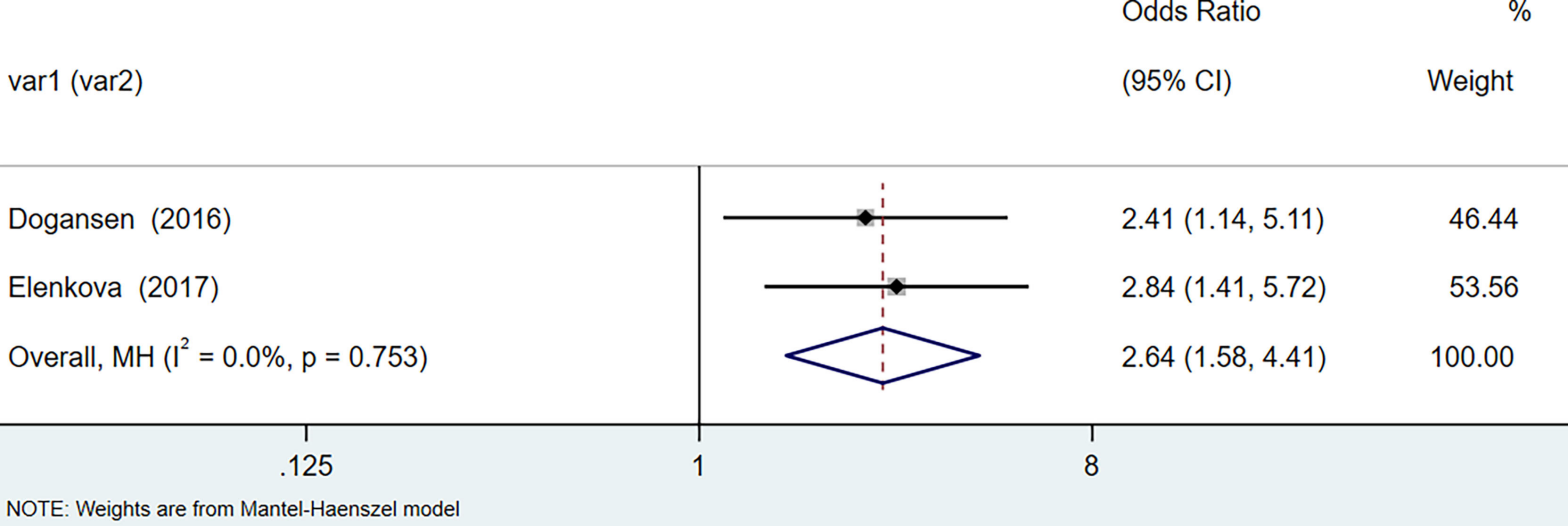
Forest plot of risk of HT in patients with prolactinoma.

The results of the meta-analysis showed that the relative risk of breast cancers among HT patients was 1.69 (95% CI = 1.30–3.20, I^2^ = 39.9%, p = 0.19) under the fixed effects model (**Figure 4**). There was no heterogeneity between studies and the findings were statistically significant. In terms of respiratory cancers, a total of two articles (30, 32) described the incidence of lung cancer in HT patients. We performed a meta-analysis using a random-effects model and RR was 2.04 (95% CI = 0.02–171.49, I^2^ = 84.2%, p = 0.012), and the results showed that patients with HT have an increased risk of developing lung cancer relative to patients without HT (**Figure 5**). Cancers of the digestive system involve gastric cancer, bowel cancer, hepatobiliary cancer, and RR was 2.84, (95% CI = 1.54–5.24, I^2^ = 0.0%, p = 0.591).The results of the study indicate that patients with HT have an increased risk of developing cancers of the digestive system, compared with patients without HT (**Figure 6**). Urogenital cancers include uterus, cervical, ovary, prostate, bladder, kidney, and the results demonstrates that RR is 1.53 (RR = 1.53, 95% CI = 0.74–3.18, I^2^ = 0.0%, p = 0.475 >0.1) (**Figure 7**). This suggests an increased risk of urogenital cancers in HT patients. Blood cancers are mainly leukemia incidence in HT patients, we performed a meta-analysis of the included literature using a fixed-effects model, and the results showed that RR is 4.11, (95% CI = 0.96–17.63, I^2^ = 0.0%, p = 0.450) (**Figure 8**). This suggests that HT may increase the risk of developing leukemia. We conducted a meta-analysis of articles investigating the relationship between prolactinoma and HT using a fixed-effects model, and the results showed that OR value is 2.64 (95% CI = 1.58–4.41, I^2^ = 0.0%, p = 0.753) (**Figure 9**). This suggests a positive correlation between HT and prolactinomas.

## 4 Discussion

The meta-analysis result of these observational studies demonstrated that people with HT were significantly associated with a high risk of thyroid cancer, breast cancers, lung cancer, digestive system cancer, urogenital cancers, blood cancers, and prolactinoma people without HT. These results are consistent with previous research findings, which found that HT patients had significantly increased risks of thyroid cancer, lung cancer, breast cancer, and leukemia (43–48). As early as 1863, Rudolf Virchow linked chronic inflammation and tumors (49). Subsequently, growing studies on the relationship between chronic inflammation and tumors supported this hypothesis (3, 50).

Although the mechanism between Hashimoto’s thyroiditis and carcinogenesis is unclear, several hypotheses have been proposed. Among all the hypotheses, chronic inflammation induces cancer as one of the possible mechanisms (51–53). Under the influence of a chronic inflammatory environment, people are more likely to switch to several types of cancer: breast, liver, bowel, bladder, prostate, gastric mucosa, ovarian and skin cancers (53). When tissue is damaged, inflammatory cells will aggregate to release reactive oxygen species (ROS) and inflammatory cytokines, and subsequently induce cell proliferation, cell repair, and the formation of a chronic inflammatory environment (3, 53). The ROS/RNS generated in the inflammatory environment can cause DNA damage in organs, which is a common mechanism of cancer development, especially 8-oxo -7,8-dihydro-2′-deoxyguanosine and 8-nitroguanidine (54). In an inflammatory environment, the generated ROS/RNS damage not only DNA but also proteins and lipids (55). NADH oxidase and iNOS in inflammatory cells can produce superoxide ${\text{O}}_{2}^{\cdot -}$ and NO, and the hydroxyl radical (•OH) generated by the reaction of H_2_O_2_ generated by O_2_ mutation with Fe (II) can attack DNA, proteins, and lipids (55). Furthermore, DNA methylation is a key factor in inflammation-induced cancer. In an inflammatory environment, DNA methyltransferase 1 (DNMT1) is affected by ROS/RNS or pro-inflammatory factors, resulting in enhanced DNA methylation of tumor suppressor genes and microRNAs (56). Many studies have shown that inflammation is inextricably linked with the occurrence of cancer, and plays a major role in the occurrence and development of cancer. HT the thyroid microenvironment is characterized by the infiltration of lymphocytes and other immune-sensing cells, including chemokines, cytokines, and growth factors, which are important components of cellular transformation and tumor progression (57, 58). This supports the possible involvement of inflammatory molecular mechanisms in tumor development.

The immune microenvironment of the thyroid is influenced by 3 factors: TSH, reactive oxygen species (ROS), and iodine (59). Studies have shown that serum TSH is closely related to the risk of thyroid cancer, and both TSH level and thyroid autoimmunity are independent risks of malignant tumors (60, 61). However, the study found that in Graves disease patients, the prevalence of papillary thyroid cancer is higher (62). In addition, it was reported that both TGAb and TPOAb were related to the occurrence of papillary thyroid carcinoma (PTC) and that TPOAb was not as correlated with PTC as TGAb (63, 64). This suggests that the occurrence of thyroid cancer is influenced by multiple factors. Some studies on HT and breast cancer suggest that anti-TSH-R autoantibodies are associated with breast cancer risk and TSH-R is present in mammary epithelial cells (65, 66). The articles we retrieved on the risk of cancer in HT patients did not describe the TSH levels in HT patients in detail, which is regrettable that the effect of TSH levels on cancer risk could not be explored by subgroup analysis according to thyroid function.

This study included many large observational studies. We performed subgroup analyses by cancer type and by country region for each study to reduce variability. The results suggest that there are differences in the risk of thyroid cancer, breast cancers, lung cancer, digestive system cancer, urogenital cancers, blood cancers, and prolactinoma between HT and non-HT patients. Most of the studies we included were related to thyroid cancer, and the number of non-thyroid cancer studies was small, which may be related to the current lack of large, high-quality studies investigating the incidence of cancer in patients with HT. Therefore, more high-quality studies are needed to document the health management of HT patients in the future for better cancer diagnosis. In addition, the relationship between HT patients and cancer occurrence found in our study can be helpful for early disease screening of HT patients.

Our research has the following strengths. We report the risk of developing multiple cancers among patients with HT and those without HT, not limited to the risk of developing thyroid cancer. We performed analyses according to different cancer types to more accurately assess the correlation between HT and cancer. However, our study also has some limitations. Firstly, despite our careful search of the database, there are still some studies that may be missed. Secondly, the meta-analysis of lung cancer risk in HT patients exist heterogeneous. This may be related to statistical differences in the study population, such as differences in region, ethnicity, lifestyle, and diagnostic methods. Lastly, most of the articles we finally included described the risk of developing cancer in HT patients, and only a few articles were observational studies grouped by TSH levels in HT patients. Unfortunately, due to the lack of these details in the included studies, we could not perform additional subgroup analyses to detect these associations. Therefore, it is hoped that there will be more high-quality studies exploring the relationship between HT patients and cancer in the future.

## 5 Conclusions

In conclusion, our meta-outcome study showed that patients with HT may have a significantly increased risk of thyroid cancer, breast cancers, lung cancer, digestive system cancer, urogenital cancers, blood cancers, and prolactinoma people without HT. Our findings suggest that patients with HT may be at increased risk of developing these cancers, but a more definitive answer needs to be based on extensive high-quality research. Regular screening of HT patients for cancer risk has clinical implications. Future studies should build more detailed models of risk factors between HT patients and cancer, such as serum TSH levels, region, ethnicity, and lifestyle. This will help us to explore the link between HT patients and carcinogenesis.

## Data Availability Statement

The original contributions presented in the study are included in the article/supplementary material. Further inquiries can be directed to the corresponding author.

## Author Contributions

XH and HQ co-designed this study. XH and XW drafted the research design. WF and YY. Search the database, delete duplicates and filter according to the search subject. XC and SZ extracted data and assessed the risk of bias. Data analysis was done by XH, XW, and YL discussed with all members. Finally, the first draft is revised by XH. All authors contributed to the article and agreed to the submitted version.

## Funding

Construction project for National Regional Chinese medicine surgery diagnosis and treatment center(2018); Construction project for Shanghai Municipal Health Commission East China Area of TCM special disease alliance(2021); Construction project for Shanghai Municipal Health Commission key clinical speciality (shslczdzk03801); Construction project for Shanghai Municipal Health Commission inheritance and innovation team of the Shanghai-style Traditional Chinese Medicine (2021LPTD-001).

## Conflict of Interest

The authors declare that the research was conducted in the absence of any commercial or financial relationships that could be construed as a potential conflict of interest.

## Publisher’s Note

All claims expressed in this article are solely those of the authors and do not necessarily represent those of their affiliated organizations, or those of the publisher, the editors and the reviewers. Any product that may be evaluated in this article, or claim that may be made by its manufacturer, is not guaranteed or endorsed by the publisher.

## References

[1] Bliddal S Nielsen CH Feldt-Rasmussen U . Recent Advances in Understanding Autoimmune Thyroid Disease: The Tallest Tree in the Forest of Polyautoimmunity. F1000Res (2017) 6:1776. doi: 10.12688/f1000research.11535.1 29043075 PMC5621109

[2] Ralli M Angeletti D Fiore M D'Aguanno V Lambiase A Artico M . Hashimoto's Thyroiditis: An Update on Pathogenic Mechanisms, Diagnostic Protocols, Therapeutic Strategies, and Potential Malignant Transformation. Autoimmun Rev (2020) 19(10):102649. doi: 10.1016/j.autrev.2020.102649 32805423

[3] Coussens LM Werb Z . Inflammation and Cancer. Nature (2002) 420(6917):860–7. doi: 10.1038/nature01322 PMC2803035 12490959

[4] Murata M . Inflammation and Cancer. Environ Health Prev Med (2018) 23(1):50. doi: 10.1186/s12199-018-0740-1 30340457 PMC6195709

[5] Balkwill F Mantovani A . Inflammation and Cancer: Back to Virchow? Lancet (2001) 357(9255):539–45. doi: 10.1016/S0140-6736(00)04046-0 11229684

[6] Reuter S Gupta SC Chaturvedi MM Aggarwal BB . Oxidative Stress, Inflammation, and Cancer: How are They Linked? Free Radic Biol Med (2010) 49(11):1603–16. doi: 10.1016/j.freeradbiomed.2010.09.006 PMC2990475 20840865

[7] Malhab LJB Saber-Ayad MM Al-Hakm R Nair VA Paliogiannis P Pintus G . Chronic Inflammation and Cancer: The Role of Endothelial Dysfunction and Vascular Inflammation. Curr Pharm Des (2021) 27(18):2156–69. doi: 10.2174/1381612827666210303143442 33655853

[8] L'Heureux A Wieland DR Weng CH Chen YH Lin CH Lin TH . Association Between Thyroid Disorders and Colorectal Cancer Risk in Adult Patients in Taiwan. JAMA Netw Open (2019) 2(5):e193755. doi: 10.1001/jamanetworkopen.2019.3755 31099862 PMC6537921

[9] Hanege FM Tuysuz O Celik S Sakallıoglu O Arslan Solmaz O . Hashimoto's Thyroiditis in Papillary Thyroid Carcinoma: A 22-Year Study. Acta Otorhinol Ital (2021) 41(2):142–5. doi: 10.14639/0392-100X-N1081 PMC8142732 34028458

[10] Uhliarova B Hajtman A . Hashimoto's Thyroiditis - an Independent Risk Factor for Papillary Carcinoma. Braz J Otorhinolaryngol (2018) 84(6):729–35. doi: 10.1016/j.bjorl.2017.08.012 PMC9442860 28964693

[11] Grani G Calvanese A Carbotta G D'Alessandri M Nesca A Bianchini M . Thyroid Autoimmunity and Risk of Malignancy in Thyroid Nodules Submitted to Fine-Needle Aspiration Cytology. Head Neck (2015) 37(2):260–4. doi: 10.1002/hed.23587 24375752

[12] Rotondi M Groppelli G Croce L Latrofa F Ancona G Coperchini F . Patients With Chronic Autoimmune Thyroiditis are Not at Higher Risk for Developing Clinically Overt Thyroid Cancer: A 10-Year Follow-Up Study. Eur J Endocrinol (2020) 183(3):317–23. doi: 10.1530/EJE-20-0350 32717718

[13] Fierabracci P Pinchera A Campani D Pollina LE Giustarini E Giani C . Association Between Breast Cancer and Autoimmune Thyroid Disorders: No Increase of Lymphocytic Infiltrates in Breast Malignant Tissues. J Endocrinol Invest (2006) 29(3):248–51. doi: 10.1007/BF03345548 16682839

[14] Stang A . Critical Evaluation of the Newcastle-Ottawa Scale for the Assessment of the Quality of Nonrandomized Studies in Meta-Analyses. Eur J Epidemiol (2010) 25(9):603–5. doi: 10.1007/s10654-010-9491-z 20652370

[15] Higgins JP Thompson SG Deeks JJ Altman DG . Measuring Inconsistency in Meta-Analyses. BMJ (2003) 327(7414):557–60. doi: 10.1136/bmj.327.7414.557 PMC192859 12958120

[16] Lau J Ioannidis JP Schmid CH . Quantitative Synthesis in Systematic Reviews. Ann Intern Med (1997) 127(9):820–6. doi: 10.7326/0003-4819-127-9-199711010-00008 9382404

[17] Siriweera EH Ratnatunga NV . Profile of Hashimoto's Thyroiditis in Sri Lankans: Is There an Increased Risk of Ancillary Pathologies in Hashimoto's Thyroiditis? J Thyroid Res (2010) 2010:124264. doi: 10.4061/2010/124264 21048834 PMC2955451

[18] Paparodis R Imam S Todorova-Koteva K Staii A Jaume JC . Hashimoto's Thyroiditis Pathology and Risk for Thyroid Cancer. Thyroid (2014) 24(7):1107–14. doi: 10.1089/thy.2013.0588 PMC4080848 24708347

[19] Büyükaşık O Hasdemir AO Yalçın E Celep B Sengül S Yandakçı K . The Association Between Thyroid Malignancy and Chronic Lymphocytic Thyroiditis: Should it Alter the Surgical Approach? Endokrynol Pol (2011) 62(4):303–8. 21879469

[20] Zhang Y Ma XP Deng FS Liu ZR Wei HQ Wang XH . The Effect of Chronic Lymphocytic Thyroiditis on Patients With Thyroid Cancer. World J Surg Oncol (2014) 12:277. doi: 10.1186/1477-7819-12-277 25179111 PMC4162966

[21] Paparodis RD Karvounis E Bantouna D Chourpiliadis C Chourpiliadi H Livadas S . Incidentally Discovered Papillary Thyroid Microcarcinomas Are More Frequently Found in Patients With Chronic Lymphocytic Thyroiditis Than With Multinodular Goiter or Graves' Disease. Thyroid (2020) 30(4):531–5. doi: 10.1089/thy.2019.0347 31950881

[22] Repplinger D Bargren A Zhang YW Adler JT Haymart M Chen H . Is Hashimoto's Thyroiditis a Risk Factor for Papillary Thyroid Cancer? J Surg Res (2008) 150(1):49–52. doi: 10.1016/j.jss.2007.09.020 17996901 PMC2575056

[23] Mazokopakis EE Tzortzinis AA Dalieraki-Ott EI Tsartsalis AN Syros PK Karefilakis CM . Coexistence of Hashimoto's Thyroiditis With Papillary Thyroid Carcinoma. A Retrospective Study. Hormones (Athens) (2010) 9(4):312–7. doi: 10.14310/horm.2002.1282 21112862

[24] Zhang Y Dai J Wu T Yang N Yin Z . The Study of the Coexistence of Hashimoto's Thyroiditis With Papillary Thyroid Carcinoma. J Cancer Res Clin Oncol (2014) 140(6):1021–6. doi: 10.1007/s00432-014-1629-z PMC11823965 24619663

[25] Matesa-Anić D Matesa N Dabelić N Kusić Z . Coexistence of Papillary Carcinoma and Hashimoto's Thyroiditis. Acta Clin Croat. (2009) 48(1):9–12. 19623865

[26] Jackson D Handelsman RS Farrá JC Lew JI . Increased Incidental Thyroid Cancer in Patients With Subclinical Chronic Lymphocytic Thyroiditis. J Surg Res (2020) 245:115–8. doi: 10.1016/j.jss.2019.07.025 31415932

[27] Zhang L Li H Ji QH Zhu YX Wang ZY Wang Y . The Clinical Features of Papillary Thyroid Cancer in Hashimoto's Thyroiditis Patients From an Area With a High Prevalence of Hashimoto's Disease. BMC Canc (2012) 12:610. doi: 10.1186/1471-2407-12-610 PMC3547693 23256514

[28] Mukasa K Noh JY Kunii Y Matsumoto M Sato S Yasuda S . Prevalence of Malignant Tumors and Adenomatous Lesions Detected by Ultrasonographic Screening in Patients With Autoimmune Thyroid Diseases. Thyroid (2011) 21(1):37–41. doi: 10.1089/thy.2010.0050 20932180

[29] Dogansen SC Selcukbiricik OS Bilir BE Yarman S . The Higher Incidence of Autoimmune Thyroid Disease in Prolactinomas Than in Somatotrophinomas. Growth Horm IGF Res (2016) 29:45–9. doi: 10.1016/j.ghir.2016.04.004 27105040

[30] Chen YK Lin CL Cheng FT Sung FC Kao CH . Cancer Risk in Patients With Hashimoto's Thyroiditis: A Nationwide Cohort Study. Br J Canc (2013) 109(9):2496–501. doi: 10.1038/bjc.2013.597 PMC3817335 24084773

[31] Silva de Morais N Stuart J Guan H Wang Z Cibas ES Frates MC . The Impact of Hashimoto Thyroiditis on Thyroid Nodule Cytology and Risk of Thyroid Cancer. J Endocr Soc (2019) 3(4):791–800. doi: 10.1210/js.2018-00427 30963137 PMC6446886

[32] Holm LE Blomgren H Löwhagen T . Cancer Risks in Patients With Chronic Lymphocytic Thyroiditis. N Engl J Med (1985) 312(10):601–4. doi: 10.1056/NEJM198503073121001 3838363

[33] Liu X Zhu L Cui D Wang Z Chen H Duan Y . Coexistence of Histologically Confirmed Hashimoto's Thyroiditis With Different Stages of Papillary Thyroid Carcinoma in a Consecutive Chinese Cohort. Int J Endocrinol (2014) 2014:769294. doi: 10.1155/2014/769294 25505911 PMC4255062

[34] Anil C Goksel S Gursoy A . Hashimoto's Thyroiditis is Not Associated With Increased Risk of Thyroid Cancer in Patients With Thyroid Nodules: A Single-Center Prospective Study. Thyroid (2010) 20(6):601–6. doi: 10.1089/thy.2009.0450 20470208

[35] Gul K Dirikoc A Kiyak G Ersoy PE Ugras NS Ersoy R . The Association Between Thyroid Carcinoma and Hashimoto's Thyroiditis: The Ultrasonographic and Histopathologic Characteristics of Malignant Nodules. Thyroid (2010) 20(8):873–8. doi: 10.1089/thy.2009.0118 20677997

[36] Consorti F Loponte M Milazzo F Potasso L Antonaci A . Risk of Malignancy From Thyroid Nodular Disease as an Element of Clinical Management of Patients With Hashimoto's Thyroiditis. Eur Surg Res (2010) 45(3-4):333–7. doi: 10.1159/000320954 21051899

[37] Konturek A Barczyński M Wierzchowski W Stopa M Nowak W . Coexistence of Papillary Thyroid Cancer With Hashimoto Thyroiditis. Langenbecks Arch Surg (2013) 398(3):389–94. doi: 10.1007/s00423-012-1021-x PMC3597286 23099542

[38] Dailey Me Lindsay S Skahen R . Relation of Thyroid Neoplasms to Hashimoto Disease of the Thyroid Gland. AMA Arch Surg (1955) 70(2):291–7. doi: 10.1001/archsurg.1955.01270080137023 13227748

[39] Elenkova A Аtanasova I Кirilov G Natchev Е Ivanova R Кovatcheva R . Autoimmune Hypothyroidism is Three Times More Frequent in Female Prolactinoma Patients Compared to Healthy Women: Data From a Cross-Sectional Case-Control Study. Endocrine (2017) 57(3):486–93. doi: 10.1007/s12020-017-1372-8 28726182

[40] McLeod MK East ME Burney RE Harness JK Thompson NW . Hashimoto's Thyroiditis Revisited: The Association With Thyroid Cancer Remains Obscure. World J Surg (1988) 12(4):509–16. doi: 10.1007/BF01655435 3420933

[41] de Matos PS Ferreira AP Ward LS . Prevalence of Papillary Microcarcinoma of the Thyroid in Brazilian Autopsy and Surgical Series. Endocr Pathol (2006) 17(2):165–73. doi: 10.1385/ep:17:2:165 17159249

[42] de Alcântara-Jones DM de Alcântara-Nunes TF Rocha Bde O de Oliveira RD Santana AC de Alcântara FT . Is There Any Association Between Hashimoto's Thyroiditis and Thyroid Cancer? A Retrospective Data Analysis. Radiol Bras (2015) 48(3):148–53. doi: 10.1590/0100-3984.2014.0072 PMC4492566 26185340

[43] Yamashita N Maruchi N Mori W . Hashimoto's Thyroiditis: A Possible Risk Factor for Lung Cancer Among Japanese Women. Cancer Lett (1979) 7(1):9–13. doi: 10.1016/s0304-3835(79)80070-1 582295

[44] Kurland LT Annegers JF . Letter: Breast Cancer and Hashimoto Thyroiditis. Lancet (1976) 1(7963):808. doi: 10.1016/s0140-6736(76)91650-0 56621

[45] Muller I Pinchera A Fiore E Belardi V Rosellini V Giustarini E . High Prevalence of Breast Cancer in Patients With Benign Thyroid Diseases. J Endocrinol Invest (2011) 34(5):349–52. doi: 10.1007/BF03347458 20595798

[46] Feldt-Rasmussen U . Hashimoto's Thyroiditis as a Risk Factor for Thyroid Cancer. Curr Opin Endocrinol Diabetes Obes (2020) 27(5):364–71. doi: 10.1097/MED.0000000000000570 32773575

[47] Dailey ME Lindsay S Skahen R . Relation of Thyroid Neoplasms to Hashimoto Disease of the Thyroid Gland. AMA Arch Surg (1955) 70(2):291–7. doi: 10.1001/archsurg.1955.01270080137023 13227748

[48] Perillat-Menegaux F Clavel J Auclerc MF Baruchel A Leverger G Nelken B . Family History of Autoimmune Thyroid Disease and Childhood Acute Leukemia. Cancer Epidemiol Biomarkers Prev (2003) 12(1):60–3. 12540505

[49] Balkwill F Mantovani A . Inflammation and Cancer: Back to Virchow? Lancet (2001) 357(9255):539–45. doi: 10.1016/S0140-6736(00)04046-0 11229684

[50] Kyewski B Romero P . Chronic Inflammation is Regarded as a Strong Promoter of Tumorigenesis. Int J Canc (2010) 127(4):747. doi: 10.1002/ijc.25487 20564540

[51] Baniyash M Sade-Feldman M Kanterman J . Chronic Inflammation and Cancer: Suppressing the Suppressors. Cancer Immunol Immunother. (2014) 63(1):11–20. doi: 10.1007/s00262-013-1468-9 23990173 PMC11029780

[52] Hussain SP Harris CC . Inflammation and Cancer: An Ancient Link With Novel Potentials. Int J Canc (2007) 121(11):2373–80. doi: 10.1002/ijc.23173 17893866

[53] Khandia R Munjal A . Interplay Between Inflammation and Cancer. Adv Protein Chem Struct Biol (2020) 119:199–245. doi: 10.1016/bs.apcsb.2019.09.004 31997769

[54] Ohnishi S Ma N Thanan R Pinlaor S Hammam O Murata M . DNA Damage in Inflammation-Related Carcinogenesis and Cancer Stem Cells. Oxid Med Cell Longev (2013) 2013:387014. doi: 10.1155/2013/387014 24382987 PMC3870134

[55] Murata M . Inflammation and Cancer. Environ Health Prev Med (2018) 23(1):50. doi: 10.1186/s12199-018-0740-1 30340457 PMC6195709

[56] Rokavec M Öner MG Hermeking H . Lnflammation-Induced Epigenetic Switches in Cancer. Cell Mol Life Sci (2016) 73(1):23–39. doi: 10.1007/s00018-015-2045-5 26394635 PMC11108555

[57] Lewinski A Sliwka PW Stasiolek M . Dendritic Cells in Autoimmune Disorders and Cancer of the Thyroid. Folia Histochem Cytobiol. (2014) 52(1):18–28. doi: 10.5603/FHC.2014.0002 24802957

[58] Pagano L Mele C Sama MT Zavattaro M Caputo M De Marchi L . Thyroid Cancer Phenotypes in Relation to Inflammation and Autoimmunity. Front Biosci (Landmark Ed). (2018) 23(12):2267–82. doi: 10.2741/4705 29772561

[59] Lun Y Wu X Xia Q Han Y Zhang X Liu Z . Hashimoto's Thyroiditis as a Risk Factor of Papillary Thyroid Cancer may Improve Cancer Prognosis. Otolaryngol Head Neck Surg (2013) 148(3):396–402. doi: 10.1177/0194599812472426 23300224

[60] Boi F Pani F Mariotti S . Thyroid Autoimmunity and Thyroid Cancer: Review Focused on Cytological Studies. Eur Thyroid J (2017) 6(4):178–86. doi: 10.1159/000468928 PMC5567004 28868258

[61] Boi F Minerba L Lai ML Marziani B Figus B Spanu F . Both Thyroid Autoimmunity and Increased Serum TSH are Independent Risk Factors for Malignancy in Patients With Thyroid Nodules. J Endocrinol Invest (2013) 36(5):313–20. doi: 10.3275/8579 22931861

[62] Kunjumohamed FP Al-Busaidi NB Al-Musalhi HN Al-Shereiqi SZ Al- Salmi IS . The Prevalence of Thyroid Cancer in Patients With Hyperthyroidism. Saudi Med J (2015) 36(7):874–77. doi: 10.15537/smj.2015.7.11463 PMC4503911 26108596

[63] Fiore E Rago T Latrofa F Provenzale MA Piaggi P Delitala A . Hashimoto's Thyroiditis is Associated With Papillary Thyroid Carcinoma: Role of TSH and of Treatment With L-Thyroxine. Endocr Relat Canc (2011) 18(4):429–37. doi: 10.1530/ERC-11-0028 21565972

[64] Kim KW Park YJ Kim EH Park SY Park DJ Ahn SH . Elevated Risk of Papillary Thyroid Cancer in Korean Patients With Hashimoto's Thyroiditis. Head Neck (2011) 33(5):691–5. doi: 10.1002/hed.21518 21484918

[65] Davies TF . The Thyrotropin Receptors Spread Themselves Around. J Clin Endocrinol Metab (1994) 79(5):1232–3. doi: 10.1210/jcem.79.5.7962313 7962313

[66] Chen YK Lin CL Chang YJ Cheng FT Peng CL Sung FC . Cancer Risk in Patients With Graves' Disease: A Nationwide Cohort Study. Thyroid (2013) 23(7):879–84. doi: 10.1089/thy.2012.0568 PMC3704114 23421548

